# Biliary Adenofibroma with Carcinoma In Situ: A Rare Case Report

**DOI:** 10.1155/2012/793963

**Published:** 2012-09-03

**Authors:** N. Thao T. Nguyen, Theresa R. Harring, Laurie Holley, John A. Goss, Christine A. O'Mahony

**Affiliations:** ^1^Department of Surgery, Baylor College of Medicine, One Baylor Plaza, Suite 404D, Houston, TX 77030, USA; ^2^Department of Pathology, St. Luke's Episcopal Hospital, 6720 Bertner Avenue, Houston, TX 77030, USA; ^3^Division of Abdominal Transplantation, Baylor College of Medicine, One Baylor Plaza, Suite 404D, Houston, TX 77030, USA

## Abstract

This case report exhibits a rare biliary tumor within the liver of a 53-year-old Caucasian woman. This exophytic, multicystic, 6.5 × 5.0 cm mass was composed of complex tubulocystic structures lined by nonmucin-secreting, biliary epithelium embedded in fibrous stroma, consistent with biliary adenofibroma. This is the seventh case described in the literature. Multiple foci of high-grade dysplasia/carcinoma in situ were found with a microscopic focus of invasive carcinoma in review of the pathology, making this only the second case reporting malignant transformation. It is presented to illustrate the premalignant potential in a biliary epithelial tumor currently categorized as benign.

## 1. Introduction

Benign biliary tumors are uncommon entities and include bile duct adenomas, von Meyenburg complexes, biliary cystadenomas, and biliary cysts that lie on a spectrum of ductal plate malformations [1]. Biliary adenofibroma (BAF) has been categorized within this group, an extremely rare entity [2] with only 6 human cases reported in English publication [3–7].

First described by Tsui et al., BAF is characterized by a proliferation of nonmucin-secreting, tubulocystic structures variably embedded in a fibrous stroma. It likely originates from interlobular or larger bile ducts based on immunohistochemistry. Though it is categorized as a benign tumor with indolent behavior, there is a concern of malignant potential [7–9]. Tsui et al. noted its “expansile growth, possession of mitoses, and foci of epithelial tufting and cellular atypia” to suggest a neoplastic process.

This case report represents the 2nd case known with evidence of high-grade dysplasia/carcinoma in situ out of seven biliary adenofibromas now reported in the current medical literature.

The significance of this case is to show biliary adenofibroma as a premalignant condition, differentiated from a benign biliary neoplasm. Despite its rarity, BAF proves to have malignant transformation in two of the seven cases now reported and warrants greater acknowledgement for this propensity.

## 2. Report of a Case

**Figure 1:**
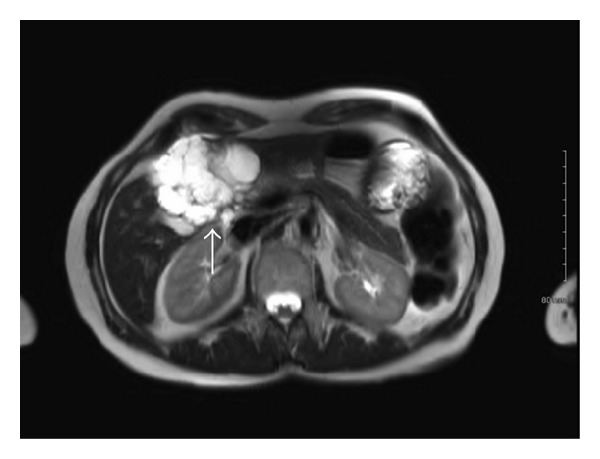
MRI image detailing a 6.6 × 5 cm cystic mass originating from segment 4B of the liver (arrow).

Our patient is a 53-year-old Caucasian female with a past medical history of Hashimoto thyroiditis, osteopenia, and uterine fibroids. Her surgical history was significant for a thyroid biopsy but otherwise, she was a healthy woman requiring no medicines. She denied any history of alcohol use, blood transfusions, intravenous drug use, tattoos, or body piercings. Her family history consisted of breast cancer and cardiac disease in her mother and a history of aortic dissection in her father. She is without any previous personal or family history of hepatobiliary disease. During her laparoscopic total abdominal hysterectomy for uterine fibroids, she was incidentally found to have a left hepatic complex cystic mass. An ultrasound of the liver revealed a 5.3 × 5.5 × 6 cm complex cystic mass concerning a neoplasm, which prompted an MRI study. MRI revealed a 6.6 × 5 cm multiseptated mass within the inferior portion of the medial segment of the left lobe, segment 4b (Figure 1). It was exophytic in nature extending inferiorly and abutting the pancreatic head. Septal enhancement was noted with gadolinium administration. Differential diagnoses included biliary cystadenoma and cystadenocarcinoma though radiographic characteristics could not confirm a diagnosis. Liver function tests and clotting profile were within normal limits. No hepatitis B surface antigen was detected. The patient had serum tumor markers drawn; she had an elevated CA-125 serum level of 64 units/mL at the time of her total abdominal hysterectomy, which normalized afterwards. AFP, CA 19-9, and CEA were all within normal limits [6]. The patient reported a 17-pound weight loss since her hysterectomy 3 months prior but denied any other associated symptoms upon review of systems. Therefore, the clinical decision was made to resect the mass for diagnosis and treatment. 

The patient was taken to an operating room for an exploratory laparotomy, adhesiolysis, cholecystectomy, and segmental resection of Couinaud's segments 3 and 4.

Her postoperative course was uneventful, and the patient was discharged on postoperative day 3. Histology of her hepatic mass was then found to be biliary adenofibroma with a microscopic focus of invasive carcinoma. Considering that only 6 cases have been reported in the literature, with only one reporting malignant transformation, clinical management for this disease process has yet to be delineated [8, 9]. With complete resection and no evidence of metastatic disease, the patient is currently managed expectantly by her oncologist. She is without evidence of recurrence 12 months after resection.

## 3. Pathology

### 3.1. Macro

The hepatic specimen consisted of a 6.5 × 5 × 3.5 cm exophytic multicystic mass located 3 mm from the closest parenchymal margin of resection. The surrounding liver appeared normal. The cysts were filled with nonmucinous fluid. The cystic lesion itself originated from Couinaud's segments 3 and 4, 3 mm from the closest parenchymal margin of resection. 

### 3.2. Micro

**Figure 2:**
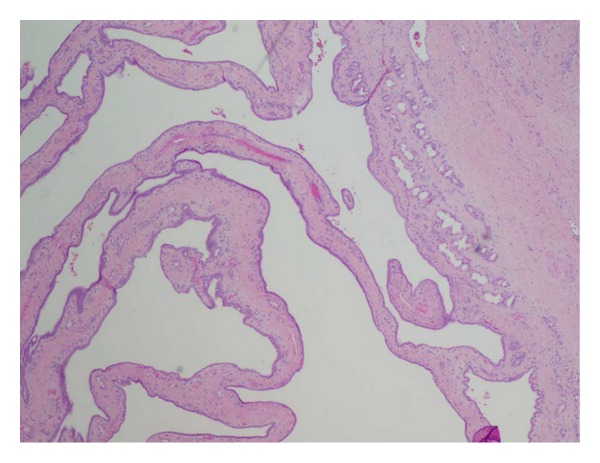
Low magnification revealing cystic tubules in a nest of fibrous stroma. Tubules are lined by cuboidal bile duct epithelium against the background of fibrous stroma.

**Figure 3:**
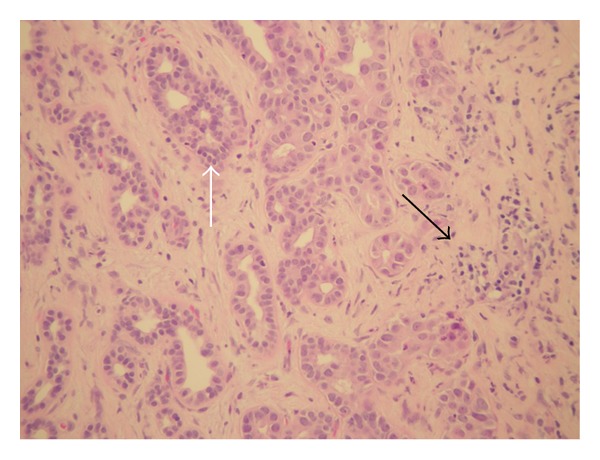
High-grade dysplasia with epithelial tufting and cellular atypia (white arrow). Microscopic focus of invasive carcinoma (black arrow).

Microscopic findings of the mass were composed of proliferation of variably sized tubulocystic structures lined by cuboidal epithelium, embedded in a fibrous stroma (Figure 2). Many areas of the lesion consisted of small tubules and cystic spaces lined by cuboidal biliary epithelium without atypia. There were multiple foci of epithelial atypia (including cytologic atypia and architectural atypia with cribriforming and a back-to-back arrangement of glands) that represented high-grade dysplasia. A small focus of invasive carcinoma was found (<1 mm), with increased atypia (including mildly enlarged epithelial cells with prominent nucleoli, a rare single cell in the stroma, and disrupted glands that merge with the sclerotic stroma) that could represent microinvasion (Figure 3). No areas of perineural or lymphovascular invasion were seen. On histochemical staining, the glandular epithelium was strongly positive for cytokeratin 7 and 19.

## 4. Comment

Biliary adenofibroma has been described in 5 women and 2 men, including this publication. This report describes the first female patient to have malignant transformation of her BAF, the second overall. There are two cases previously reported of malignant transformation [8, 9], though these cases reported a male patient in his twenties with a primary tumor measuring 20 cm, originating from the same medical center in Turkey. One of these reports was found to have recurrence with metastatic disease in his lungs after primary resection of his 20 cm mass three years prior. Considering the extreme rarity of the disease, the clinical history of both men and timing of the case reports, it is highly likely these are two different reports of the same man, at varying points of his disease progression (O. Akin, personal communication, February 24th, 2012).

Regardless, BAF is currently described as a benign entity [3], though now, including this report, two of the 7 cases in the literature present with malignant transformation. Redefining this rare process from a benign to a premalignant process alerts clinicians to be wary of its malignant potential, enabling afflicted patients to be more vigilantly followed with interventions implemented earlier in the course of their disease. Presumably, early recognition of recurrence could improve prognosis. Accurate identification and characterization of biliary adenofibroma, likely a premalignant disease process, is necessary to aid in management.
